# Recombinant Nonstructural 3 Protein, rNS3, of Hepatitis C Virus Along With Recombinant GP96 Induce IL-12, TNFα and α5integrin Expression in Antigen Presenting Cells

**DOI:** 10.5812/hepatmon.8104

**Published:** 2013-06-03

**Authors:** Mohammad Reza Hajizadeh, Pooneh Mokarram, Eskandar Kamali sarvestani, Azam Bolhassani, Zohreh Mostafavi Pour

**Affiliations:** 1 Recombinant Proteins Lab, Biochemistry Department, Medical School, Shiraz University of Medical Sciences, Shiraz, IR Iran; 2 Gastroentrohepatology Research Center, Medical School, Shiraz University of Medical Sciences, Shiraz, IR Iran; 3 Immunology Department, Medical School, Shiraz University of Medical Sciences, Shiraz, IR Iran; 4 Molecular Immunology and Vaccine Research Laboratory, Pasteur Institute of Iran, Tehran, IR Iran; 5 Faculty for Advanced Biomedical Sciences, Shiraz University of Medical Sciences, Shiraz, IR Iran

**Keywords:** Hepatitis C, Cytokines, Heat- Shock Proteins

## Abstract

**Background:**

Hepatitis C virus (HCV) infection is the main cause of chronic liver disease and to date there has been no vaccine development to prevent this infection. Among non-structural HCV proteins, NS3 protein is an excellent goal for a therapeutic vaccine, due to its large size and less variation in conserved regions. The immunogenic properties of heat shock proteins (HSPs) for instance GP96 have prompted investigations into their function as strong adjuvant to improve innate and adaptive immunity.

**Objectives:**

The aim of this study was to examine additive effects of recombinant GP96 (rGP96) fragments accompanied by rNS3 on expression levels of α5integrin and pro-inflammatory cytokines, IL-12 and TNFα, in Antigen Presenting Cells (APCs).

**Materials and Methods:**

Recombinant viral proteins (rNS3 and rRGD-NS3), N-terminal and C-terminal fragments of GP96 were produced and purified from *E. coli *in order to treat the cells; mouse spleen Dendritic Cells (DCs) and THP-1 macrophages.

**Results:**

Our results showed that rNT-GP96 alone significantly increases the expression level of IL-12, TNFα and α5integrin in THP-1 macrophages and DCs, while IL-12 and TNFα expression levels were unaffected by either rNS3 or rRGD-NS3. Interestingly, the co-addition of these recombinant proteins with rNT-GP96 increased IL-12, TNFα and α5integrin expression. Pearson Correlation showed a direct association between α5integrin with IL-12 and TNF-α expression.

**Conclusions:**

we have highlighted the role of rNS3 plus rNT-GP96 mediated by α5integrin in producing IL-12 and TNFα. It can be suggested that rNT-GP96 could enhance immunity characteristic of rNS3 protein via production of pro-inflammatory cytokines.

## 1. Background

Hepatitis C virus (HCV) infection is considered as one of the main causes of chronic liver diseases. Approximately 130 to 170 million people are chronically infected by HCV and this can lead to end-stage liver disease (ESLD) and liver failure (1, 2). It is estimated that 3 - 4 million people are infected with HCV and annually more than 350,000 patients die from HCV-related liver diseases, worldwide (3, 4). According to the latest reports 1a is the most frequent sere variant of HCV in Iran (5, 6). Notably, no vaccine exists to prevent HCV infection (7) and the most effective current clinical treatment is a combination therapy with interferon alpha and ribavirin which is effective in just 40 - 50% of HCV subtype 1a (8). Recent promising data indicate that the development of directly acting antiviral (DAA) therapies including compounds targeting viral attachment and entry, nucleoside or non-nucleoside analogs, NS3-4A protease inhibitors, NS5A polymerase inhibitors and NS5A inhibitors will likely be a very potent option for patients suffering HCV (8, 9). Currently, DAA combination treatment regimens appear to be a major breakthrough in the treatment of patients with HCV subtype 1a with limited sensitivity to interferon alpha. However to achieve the successful use of these drugs, careful monitoring of HCV viral load, antiviral resistance, extra side effects and possible drug-drug interactions will be required. Therefore, it is necessary to have advancements in therapeutic procedures for prevention and treatment of HCV infections. Since some patients with chronic infections have fewer responder T-cell, favorable treatments are required for augmentation of cellular immunity on the basis of T-cell intensification (10). The whole HCV genome encodes a precursor polyprotein containing approximately 3000 amino acids which is composed of both structural (core and envelope proteins E1, E2) and non-structural proteins (NS2, NS3, NS4A, NS4B, NS5A and NS5B) (11). The NS3 gene contains less variable regions than other HCV genes and due to its large size; NS3 is an excellent goal for development of a therapeutic vaccine. Furthermore, a strong correlation exists between NS3 specific T cell responses and viral clearance of acute infection. However T-cell responses have not been found in chronic infection (12). Antigens show low immunogenicity, unless accompanied by adjuvants which introduce them to the Antigen Presenting Cells (APCs) for secretion of pro-inflammatory cytokine (IL-12 and TNF-α). These cytokines stimulate Th1 cell differentiation, initiating an immunogenic responses (13). Heat Shock Proteins (HSPs) act as chaperones and adjuvant as well as involve in signaling process (14, 15). Among HSPs, GP96 and HSP70 have been shown to induce specific immunity responses in a manner similar to immunologic adjuvant (16, 17). The GP96 is a 96-kDa glycoprotein that is converted to phosphoprotein, mediated by casein kinase ІІ. It binds to peptide antigens to facilitate their uptake by the professional antigen-specific cells which play a fundamental part in both innate and adaptive immunity (14). Several pathogens such as bacteria, viruses and fungi activate APCs either by direct binding to the integrins or indirectly via attaching to extra cellular matrix (ECM) proteins like fibronectin (FN). Many studies have shown that integrins in cross talk with immunoreceptors such as Toll like receptors (TLR) elicit integrin mediate signaling. HCV-NS3 protein triggers TLR-specific cellular activation and induces inflammation pathways (18). Integrins mediate interactions between cells and ECM proteins. Most of the twenty four known integrins are expressed on immune cell surfaces and eight of them such as α5integrin bind to the RGD motif which is present in FN (19). A recent study reported that maturation and activation of dendritic cells (DCs) is modulated by a RGD peptide density gradient via αvintegrins (20). It has also been demonstrated that the immune response which is required for the clearance of viremia during acute HCV infection, is frequently directed against nonstructural proteins such as NS3. Bolhassani et al. (21) revealed that GP96 enhances immunogenicity of the E7 protein in the human papiloma virus. It has also been confirmed that the GP96 fragments are better preferred for application in immunization processes (22, 23). In order to investigate whether GP96 fragments in combination with HCV-NS3 could improve immune responses, we cloned, expressed and purified recombinant proteins comprised of rNS3, rRGD-NS3, and the two fragments of GP96 (rNT-GP96 and rCT-GP96). We also examined the expression of IL-12 and TNFα, in APCs treated with rGP96 fragments along with rNS3 or rRGD-NS3, in APCs.

## 2. Objectives

The aim of this study was to investigate the link between the expression levels of α5integrin and aforementioned cytokines.

## 3. Materials and Methods

### 3.1. Materials

THP-1 was obtained from the National cell bank of Iran (Pasteur Institute, IRAN). Cell culture medium, penicillin, streptomycin medium supplement, glutamine and fetal bovine serum were obtained from Gibco Life Technologies (UK). Tripure Isolation Reagent and Western blotting materials were purchased from Roche Applied Sciences (USA). cDNA Synthesis Kit was purchased from Fermentas, EU. SYBR green DNA PCR Master Mix was purchased from the Applied Biosystem (ABI) Company, (Foster City, CA USA). A microbead (MACS) was obtained from Miltenyi Biotec GmbH, (Germany). Ni-NTA affinity column was obtained from Qiagen (Germany). Horseradish peroxidase (HRP)-conjugated, anti-polyHis Antibody, Phorbol 12-myristate13-acetate (PMA), Dimethyl sulfoxide (DMSO) and all other chemicals were from Sigma-Aldrich (USA)

### 3.2. Cloning of HCV-NS3 and NS3 Plus RGD Sequences in a Prokaryotic Expression Vector

The coding sequence of modified NS3, 195 first amino acids of the N-terminal region of HCV-NS3 and NS3 plus the RGD sequence were designed. PCR products were obtained and cloned into the pCR2.1 vector with the cloning sites of 5' HindIII and 3' BamHI (Eurofins MWG, Germany). The sequences above, NS3 and NS3 plus RGD, were then sub-cloned in to the pQE30 expression vector and sequenced (Eurofins MWG, Germany). pQE30-NT-GP96 and pQE30-CT-GP96 plasmids were kindly provided by Dr. Rafati (Pasteur Institute, Iran).

### 3.3. Expression of Recombinant Proteins

Following transformation of pQE30-NS3, pQE30-RGD-NS3, pQE30-NT-GP96 & pQE30-CT-GP96 plasmids to the expression host of *E. coli *M15, several colonies containing the recombinant plasmids were grown in Luria Bertani medium (LB) supplemented with ampicillin [(100 µg/ml) and kanamycin (25 µg/ml)]. When the bacterial cells reached mid-log growth (OD600 measurements of 0.6 - 0.8), the expression of recombinant proteins were induced by addition of isopropyl-D-thiogalacto-pyranoside (IPTG) to a final concentration of 1 mM and incubation continued at 37 ˚C for 4 h. Bacterial cells were collected by centrifugation (10,000 g for 5 min) and the cell pellets were further subjected to sodium dodecyl sulfate polyacrylamide gel electrophoresis (SDS-PAGE) to analyze the expression of the recombinant proteins (23).

### 3.4. SDS-PAGE and Western-Blotting

Expression of recombinant proteins was evaluated using SDS-PAGE and Western-blotting techniques. For this reason, recovered bacterial pellets before and after IPTG induction were dissolved in an appropriate volume of Laemli buffer and proteins were resolved on a 12.5% SDS-polyacrylamide gel. Western-blotting was performed based on the identification of histidine-tag in the protein. Accordingly, the SDS-PAGE separated proteins were electrotransferred onto a polyvinylidene difluoride (PVDF) membrane. Following the treatment of the blotted membrane with 1:2000 dilution of horseradish peroxidase (HRP)-conjugated anti-polyHis antibody, specific proteins were detected using an enhanced chemiluminescence (ECL) solution.

### 3.5. Purification of Recombinant Proteins

The recombinant proteins were purified based on the presence of 6xHis-tag at the N-terminus through the Ni-NTA affinity column under denaturing and native conditions. To purify the protein in the large scale, M15 *E. coli* cells expressing the recombinant plasmids were grown and induced in a 400 ml culture. The final bacterial cell pellet was resuspended in 5 ml of denaturing lysis buffer (8 M urea, 50 mM Na2HPO4, 300 mM NaCl, 10 mM imidazole, pH 8.0). The cells were further lysed by sonication (30 s pulses at 20 s intervals for six times). After loading of the lysates on the Ni-NTA column and extensive washing steps, recombinant proteins were eluted with elution buffer (8 M urea, 50 mM Na2HPO4, 300 mM NaCl, 250 mM imidazole, pH 8.0). The proteins were dialyzed in dialysis Phosphate buffered saline for 48 h, with change of buffer every 12 h to remove the urea and imidazole. The purity of the recombinant proteins was then evaluated on SDS-PAGE. To refold the purified denatured proteins, dialysis was additionally performed in 500 ml of freshly made 0.01 M phosphate buffered saline (PBS) containing gradient decreasing concentrations of 6, 4, 2, 1, 0.5, and 0 M urea in 5 mM Tris (pH 7.4) for 15 h at 4 ˚C.

### 3.6. MTT Assay

MTT assay depends on the ability of viable cells to metabolize MTT, a water-soluble tetrazolium salt, into a water-insoluble formazan product by mitochondrial succinate dehydrogenase (23). Briefly 7x10^4^ cells per well were seeded on a 96-well plate at a final volume of 200 μL. Following the cell treatments, the supernatant was replaced with 200 μL of warm RPMI 1640. After adding 10 μL of 5 mg/mL MTT to each well, the plate was incubated at 37 ˚C for 3.5 h in the dark until purple precipitate was visible under the light microscope. Then, 100 μL of DMSO was added to each well and after 15 min the absorbance was read at 570 nm with a reference filter of 620 nm.

### 3.7. Cell Line

The human monocytic cell line, THP-1, was cultured in RPMI 1640 medium supplemented with 10% fetal bovine serum and cultured at 37 ˚C in a humidified 5% (v/v) CO2 incubator. To induce differentiation, 0.5 x 10^6^ cells/well were cultured in a 24-well plate in the presence of 200 μL of growth medium supplemented with 5nM phorbol 12-myristate 13-acetate (PMA) for 48 h, medium was replaced with serum-free RPMI medium and the cells were treated with recombinant proteins for 24 h. At the end of the incubation, cell pellets were used for RNA isolation.

### 3.8. Mouse Splenic Dendritic Cell Isolation

Splenic tissue was dissociated mechanically using a scalpel and single cell suspensions were prepared by enzymatic disaggregation with collagenase D. Splenocytes were labeled with mouse CD11c+DC micro beads according to the manufacturer’s guidelines. A single cell suspension was generated and CD11c+cells were enriched using a modified MACS magnetic separation protocol as previously described (24). Purity of ~95 – 99% was detected by flow cytometry and cells were treated with recombinant proteins and GM-CSF, 10 ng/mL for 24 h.

### 3.9. Total RNA Extraction and cDNA Synthesis

Total RNA was extracted from cell cultures, DCs and THP-1 macrophages, after 24 h recombinant protein treatments by Tripure Isolation Reagent, according to manufacturer’s instructions. The quality of RNA was evaluated by measuring the ratio of absorbance at 260 nm to absorbance at 280 nm. The integrity of RNA was verified using formaldehyde gel electrophoresis by the presence of two rRNA bands (28S and 18S). First strand complementary DNA (cDNA) synthesis was performed from total RNA using Revert Aid First Strand cDNA Synthesis Kit.

### 3.10. Real-time RT-PCR

Real-time RT-PCR was carried out using the ABI real time PCR 7500 system with a two-step method. The PCR reaction mixture contained 2 μL of cDNA (tenfold diluted), 0.5μL of 5 mmol/L solutions of each of the forward and reverse primers, and 12.5 μL of SYBR green DNA PCR Master Mix in a total volume of 25 μL. Samples were loaded in duplicates. All incubations included an initial denaturation step at 95 ˚C for 10 min and 40 cycles (15 s at 95˚C and 30 s at 60˚C) subsequently. A melting curve analysis was achieved by performing 70 cycles of 10 seconds with a temperature increment of 0.5˚C/cycle starting from 60˚C. The primer sequences used for amplifications are seen in Table 1. Efficiency of amplification was measured by the slope of a standard curve, derived from tenfold dilutions of pooled cDNA. In all cases, the amplification efficiency was between 97% and 102%. Data were analyzed by using the 7500 Software v2.0.1. The relative expression level (fold changes) of genes in THP-1 and DCs were calculated by the 2 - ΔΔCT method ( 25 ).

**Table 1.** Real-time PCR Primers

Gene	Sequence of primer pair	Product Length
**β actin, mouse**	Forward: CCACACCCGCCACCAGTTCG	138
	**Reverse: CTAGGGCGGCCCACGATGGA**	
**β actin, Human**	Forward: GCTGTGCTACGTCGCCCTG	61
	**Reverse: GGAGGAGCTGGAAGCAGCC**	
**α5Integrin, Human**	Forward: TGCAGTGTGAGGCTGTGTACA	88
	**Reverse: GTGGCCACCTGACGCTCT**	
**α5Integrin, mouse**	Forward: CTGTCCGCCACTCAAGAG	215
	**Reverse: ACGGTGACATAGCCATAGG**	
**TNF-α, mouse**	Forward: GTCTCAGCCTCTTCTCATTC	99
	**Reverse: GGAACTTCTCATCCCTTTGG**	
**TNF-α, Human**	Forward: GAGTCTGGGCAGGTCTAC	199
	**Reverse: CGAAGTGGTGGTCTTGTTG**	
**IL-12, mouse**	Forward: CTTAGCCAGTCCGAAACCT	123
	**Reverse: TTGGTCCCGTGTGATGTCT**	
**IL-12, Human**	Forward: CTCCTGGACCACCTCAGTTTG	76
	**Reverse: GGTGAAGGCATGGGAACATT**	

### 3.11. Statistical Analyses

SPSS software v.17.0 was used for all statistical analysis. The statistical difference was analyzed using the one-way ANOVA. A statistically significant difference was considered if P ≤ 0.05. Data are presented as mean ± SEM. The Pearson’s r rank correlation coefficients were used to evaluate relationships between variables.

## 4. Results

### 4.1. Cloning and Expression of the Recombinant Proteins

**Figure 1.**
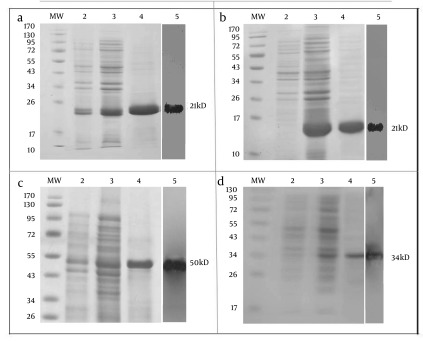
Expression and Purification of rNS3 (a), rRGD-NS3 (b), rNT-GP96 (c) and rCT-GP96 (d) in E. coli M15 Strain on SDS-PAGE 12.5%. Protein MW Marker in kDa (MW), Bacterial Extract Before Induction by IPTG (Lane 2), Crude Bacterial Lysate 4 h After Induction (Lane 3), Recombinant Proteins Purified From Bacterial Lysate by Affinity Chromatography Using Ni-NTA Resin (Lane 4) and Western Blot Assay of Recombinant Protein With His-Tag Monoclonal Antibody (lane 5).

PCR2.1-NS3 and pCR2.1-RGD-NS3 fusion proteins were cloned into pQE30 then accompanied by two terminal fragments of Gp96 (NT-GP96 and CT-GP96) in pQE30 vector, were expressed. Recombinant proteins were purified from the cell-free supernatant by affinity chromatography on Ni-NTA agarose. The expressed products were subjected to SDS-PAGE analysis using Mini Format Vertical Electrophoresis Gels run in glycin-Tris running buffer. Expression and purification results for recombinant proteins are shown in Figure 1. The theoretical molecular weights for the rNS3 and rRGD-NS3 were approximately 21kD (Figure 1 a and 1b) while the rNT-GP96 and rCT-GP96 proteins migrate as 50 and 34 kDa respectively in an *E. coli* expression system (Figure 1 c and 1d). Anti-His-antibody was used in the western blotting assay to confirm the expression of His-tag recombinant proteins (Figure 1).

### 4.2. Effects of the Recombinant Proteins on Viability of APCs

**Figure 2.**
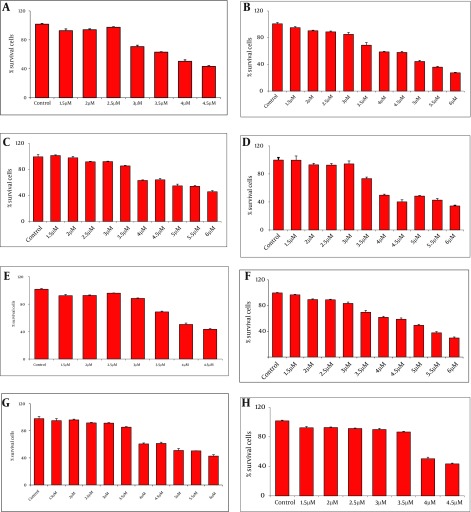
The Viability of Differentiation of THP-1 (A-D) and Dendritic Cells (E-H) in the Presence of NS3 (A and E), RGD-NS3 (B and F), CT-GP96 (C and G) and NT-GP96 (Dand H) Recombinant Proteins Evaluated by MTT Assay. Values Have Been Normalized for the Control (THP-1 and Dendritic Cells in the Absence of Recombinant Proteins), Considered as 100%, and Are the Mean of two Independent Experiments Done in Quotient.

MTT analysis at OD490 was performed 24 h post treatment of DCs and THP-1 cells with various concentrations of recombinant proteins (1.5 - 6 µM). The highest viabilities of cells were observed in the presence of 3µM rNT-GP96 and rCT-GP96 while the most suitable concentration was 2.5µM for rNS3 and rRGD-NS3 individually (Figure 2).

### 4.3. Effects of Recombinant Proteins on Expression of TNF-α, IL-12p35 and α5integrin by Real-time RT-PCR

Innate immune cells, particularly APCs play a critical role in recognition of viral pathogens via production of IL-12 and TNF-α as pro-inflammatory cytokines. In order to investigate whether recombinant proteins could activate human macrophages, DCs and THP-1 macrophages were treated for 24 h at 37 ˚C with LPS (positive control for APCs activation) and rNS3 (3 µM), rRGD-NS3 (3 µM) alone or in combination with rGP96 fragments (2.5 µM). We compared the expression levels of IL-12 and TNFα in THP-1 macrophages in the presence or absence of the recombinant proteins.

**Figure 3.**
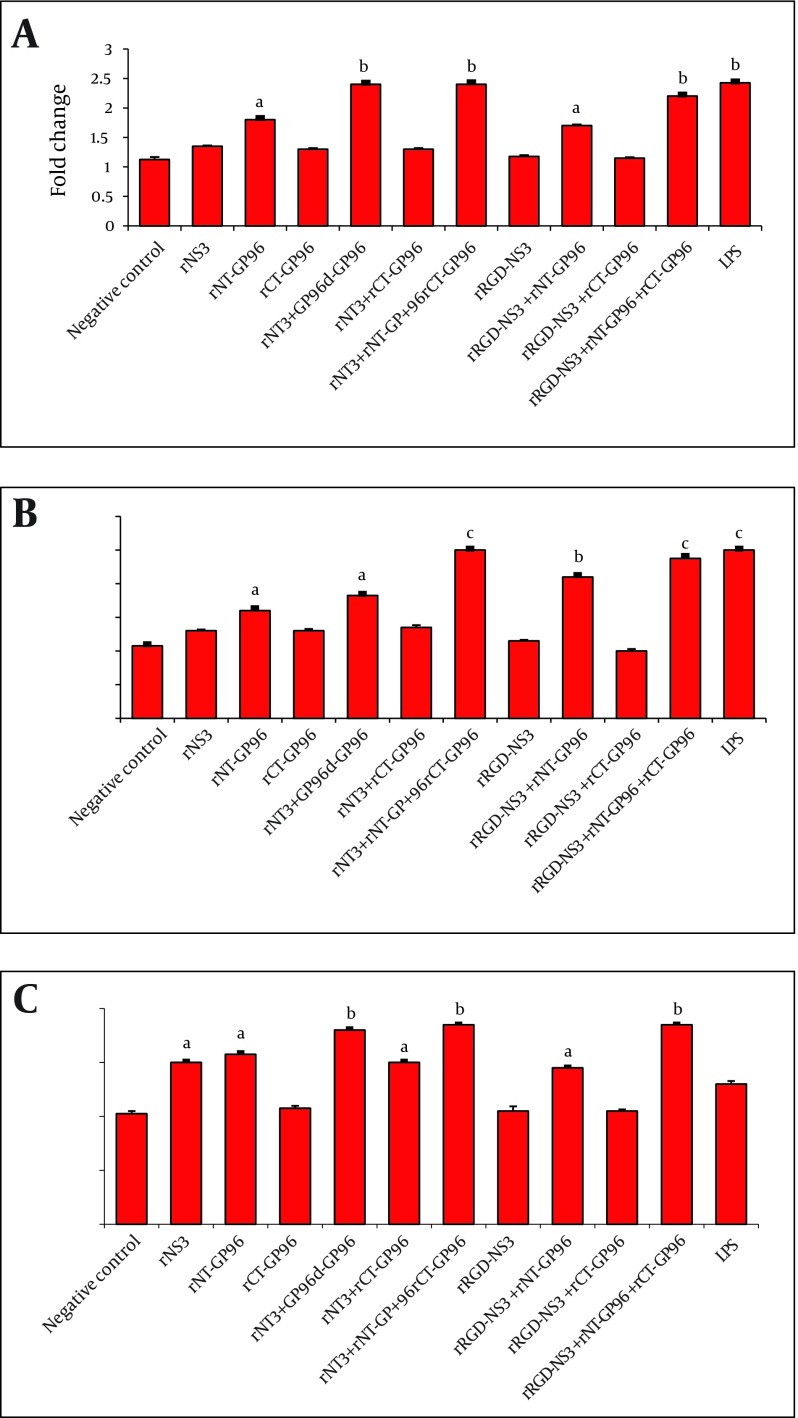
Expression of IL-12 (A), TNFα (B) and α5integrin (C) in THP-1 Macrophages Treated With Recombinant Proteins. The Cells Were Cultured for 24 h With rNS3 (3µM), rRGD-NS3 (3µM) Alone or in Combination With rGP96 Fragments (2.5µM) and LPS (1 µg/mL). The mRNA Expression Was Measured by Real-time PCR and Analyzed With the 2 - ΔΔCT Method. Comparisons Between Treated and Control Cells Were Performed Using a One-way ANOVA a P < 0.05, b P < 0.01 and c P < 0.001

**Figure 4.**
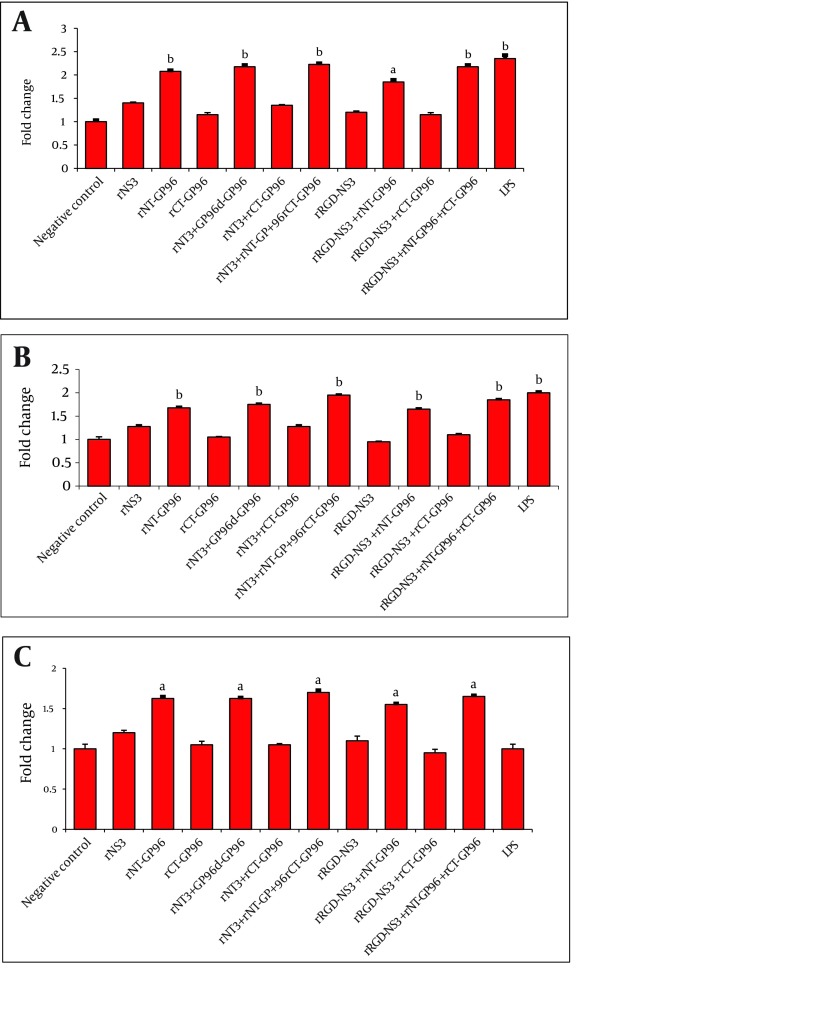
Expression of IL-12 (A), TNFα (B) and α5integrin (C) in DCs Treated With Recombinant Proteins. DCs Were Cultured for 24 h With rNS3 (3µM), rRGD-NS3 (3µM) Alone or in Combination With rGP96 Fragments (2.5µM) and LPS (1 µg/mL). The mRNA Expression Was Measured by Real-time PCR and Analyzed With the 2 - ΔΔCT Method. Comparison Between Treated and Control Cells Were Performed Using a One-way ANOVA. P < 0.05, b P < 0.01 and c P < 0.001

The rNT-GP96 alone significantly increased the expression of IL-12 mRNA (1.8-fold), while IL-12 expression level was unaffected by either rNS3 or rRGD-NS3 on their own (Figure 3 A). Interestingly the co-addition of these recombinant proteins with rNT-GP96 increased IL-12 expression (Figure 3 A). As shown in Figure 4 A, the same results were determined when we had similar treatments for DCs, as key regulator of the immune system. As we expected, treatment of THP-1 macrophages and DCs with recombinant proteins, up-regulated expression level of TNFα (Figure 3 B, 4B). When we only treated the cells with rNT-GP96, expression level of TNFα was significantly increased both in THP-1 macrophages and DCs (1.6 and 1.7-fold respectively). However the additive effect of increasing of TNFα was seen when the cells were treated with the combination of rGP96 fragments and the rNS3 (2.7-fold). This increase was comparable with LPS treated cells as positive controls (Figure 3 B, 4B). Since APC activation via αvintegrins that bind to the RGD peptide motif was already reported (19), we examined weather the increasing levels of IL-12 and TNFα in APCs treated with recombinant proteins could mediate alterations in α5integrin level of expression. In this regard rRGD-NS3 was studied with greater emphasis. Our results showed that rNT-GP96 significantly increased mRNA level of α5integrin in THP-1 macrophages and DCs (P < 0.05). Its additive effect when combined with rNT, rCT-GP96 and rNS3 was determined only in treated THP-1 macrophages (P < 0.01) (Figure 3 C, 4C). It seems that α5integrin expression might correlate with IL-12 and TNF-α expression. Pearson Correlation showed a direct association between α5integrin with IL-12 and TNF-α expression (r = 0.65 and r = 0.75, P < 0.001 respectively) in THP-1 cells. The same results were obtained from treated DCs (r = 0.66 and r = 0.73, P < 0.001 respectively).

## 5. Discussion

APCs are key regulators of the immune system, leading to immunogenic responses against antigens. Pro-inflammatory cytokines such as IL-12 and TNFα are produced mainly by activated APCs in response to stimulation by viral antigens. These cytokines perform a fundamental role in the link between innate and adaptive immune responses. HCV-NS3 has attracted considerable attention as a consequence of specific immune responses against HCV, using various adjuvants to enhance the limited immunogenicity of antigens. Therefore, according to aforementioned documented comments, our main goals for the present study could be categorized as below:

1) To express and purify the recombinant proteins containing the 195 first amino acids from HCV-NS3 alone or fused to RGD and two GP96 fragments (rNT-GP96 and rCT-GP96).

2) To evaluate the regulatory effects of these recombinant proteins on the expression of the two main pro-inflammatory cytokines i.e., IL-12 and TNFα by APCs.

3) To clarify whether the recombinant proteins treatment influences the expression of α5integrin as an antigen recognition receptor by THP-1 and DCs.

According to our results, the expression of IL-12 and TNFα was significantly higher in rNT-GP96-treated cells in comparison with the negative controls (Figure 3 and Figure 4). This part of our study is consistent with previous reports ( 21 , 26 ). The additive effects of the combined treatment of the cells with rNT-GP96 and rNS3 were detected in the expression of cytokines. Notably, when rCT-GP96 was added to the treated cells with rNT-GP96 and rNS3, no further effect was seen. Previous studies have shown the effectiveness of rNT-GP96 but not rCT-GP96 in enhancing humoral immune responses ( 27 , 28 ).

The authors demonstrated the adjuvant effect of rNT-GP96 to be the same as GP96. Mansila et al. have shown that treatment of DCs and THP-1 cells, with the fusion protein EDA-rNS3 which contain the extra domain A of FN, enhance the production of IL-12 and TNFα ( 29 ). However neither in their results nor ours the level of cytokines were significantly increased. It has been shown that several pathogens are able to bind integrin receptors on various types of host cells directly via some specific adhesion, or indirectly by using ECM proteins ( 18 ).

However, little is known about which integrins and signaling intermediates are involved. Therefore, in the present study we emphasized the importance of α5integrin in crosstalk with the pro-inflammatory cytokines of APCs. According to our results, the level of α5integrin expression was significantly up regulated in the cells treated with rNT-GP96 and rNS3 and this was comparable to the elevation of IL-12 and TNFα. This confirms molecular mechanisms suggested by Ulanova et al. in which intracellular signaling pathways were activated by bacterial engagement of integrin receptors ( 18 ). This signaling cascade leads to phosphorylation and activation of FAK and p38 and a subsequent nuclear translocation of NF-κB followed by gene expression of pro-inflammatory cytokines ( 29 ). According to our results IL-12 and TNFα may up regulate α5integrin expression through the NF-κB signaling pathway, which is consistent with the data from the Li et al. study, showing enhancement of α5integrin mediated by TNFα ( 30 ). In conclusion, we have highlighted the role of rNS3 plus rNT-GP96 mediated by α5integrin in producing IL-12 and TNFα. Here, we have emphasized that the rNS3 plus rNT-GP96 induce immune responses via enhancing IL-12 and TNFα in APCs. Therefore, these results suggest that, rNT-GP96 plus rNS3 potentiates the immune response by providing both antigen and adjuvant simultaneously to the APCs. However, further animal studies should be done to provide a novel vaccination strategy against HCV that ultimately could be applicable to humans.
